# Cytotoxic effector functions of T cells are not required for protective immunity against fatal *Rickettsia typhi* infection in a murine model of infection: Role of T_H_1 and T_H_17 cytokines in protection and pathology

**DOI:** 10.1371/journal.pntd.0005404

**Published:** 2017-02-21

**Authors:** Kristin Moderzynski, Liza Heine, Jessica Rauch, Stefanie Papp, Svenja Kuehl, Ulricke Richardt, Bernhard Fleischer, Anke Osterloh

**Affiliations:** 1 Department of Immunology, Bernhard Nocht Institute for Tropical Medicine, Hamburg, Germany; 2 Institute for Immunology, University Medical Center Hamburg-Eppendorf, Hamburg, Germany; Mahidol University, THAILAND

## Abstract

Endemic typhus caused by *Rickettsia* (*R*.) *typhi* is an emerging febrile disease that can be fatal due to multiple organ pathology. Here we analyzed the requirements for protection against *R*. *typhi* by T cells in the CB17 SCID model of infection. BALB/c wild-type mice generate CD4^+^ T_H_1 and cytotoxic CD8^+^ T cells both of which are sporadically reactivated in persistent infection. Either adoptively transferred CD8^+^ or CD4^+^ T cells protected *R*. *typhi*-infected CB17 SCID mice from death and provided long-term control. CD8^+^ T cells lacking either IFNγ or Perforin were still protective, demonstrating that the cytotoxic function of CD8^+^ T cells is not essential for protection. Immune wild-type CD4^+^ T cells produced high amounts of IFNγ, induced the release of nitric oxide in *R*. *typhi*-infected macrophages and inhibited bacterial growth *in vitro* via IFNγ and TNFα. However, adoptive transfer of CD4^+^IFNγ^-/-^ T cells still protected 30–90% of *R*. *typhi*-infected CB17 SCID mice. These cells acquired a T_H_17 phenotype, producing high amounts of IL-17A and IL-22 in addition to TNFα, and inhibited bacterial growth *in vitro*. Surprisingly, the neutralization of either TNFα or IL-17A in CD4^+^IFNγ^-/-^ T cell recipient mice did not alter bacterial elimination by these cells *in vivo*, led to faster recovery and enhanced survival compared to isotype-treated animals. Thus, collectively these data show that although CD4^+^ T_H_1 cells are clearly efficient in protection against *R*. *typhi*, CD4^+^ T_H_17 cells are similarly protective if the harmful effects of combined production of TNFα and IL-17A can be inhibited.

## Author summary

*Rickettsia typhi* causes endemic typhus, a relatively mild disease. However, complications such as encephalitis, hepatitis, pneumonia and others can occur in severe cases. In immunodeficient CB17 SCID mice *R*. *typhi* causes a 100% fatal infection and we show here that adoptively transferred CD8^+^ as well as CD4^+^ T cells that acquire a T_H_1 phenotype protect these animals from severe disease and death. We further analyzed the requirements for T cell-mediated protection and found that the cytotoxic function of CD8^+^ T cells is not essential for protection and that the release of IFNγ by these cells is more critical than the cytolytic activity for long-term control of the bacteria. Surprisingly, CD4^+^ T cells that lack IFNγ still protect 30–90% of *R*. *typhi*-infected CB17 SCID mice. These cells show a T_H_17 phenotype, producing high amounts of IL-17A and IL-22 in addition to TNFα. In *R*. *typhi*-infected CB17 SCID mice that receive IFNγ-deficient CD4^+^ T cells the neutralization of either TNFα or IL-17A mitigates disease and leads to enhanced survival, demonstrating harmful effects of these cytokines although both of them may contribute to bacterial elimination along with IL-22. We conclude that CD4^+^ T_H_17 cells are sufficient to protect against *R*. *typhi* if the detrimental effects of either TNFα or IL-17A can be inhibited. This is the first report demonstrating protection against an obligate intracellular bacterium by CD4^+^ T_H_17 cells.

## Introduction

Rickettsioses are emerging febrile diseases that can be fatal and are caused by obligate intracellular bacteria of the family of *Rickettsiaceae*. This family consists of two genera: *Orientia* with only one member (*Orientia tsutsugamushi*) and *Rickettsia* (*R*.) that is further subdivided into four groups: The spotted fever group (SFG) that contains the vast majority of rickettsiae (e.g. *R*. *rickettsii*, *R*. *conorii*), the typhus group (TG; *R*. *prowazekii* and *R*. *typhi*), the transitional group (*R*. *felis*, *R*. *akari* and *R*. *australis*) and the non-pathogenic ancestral group (*R*. *bellii* and *R*. *canadensis*) [1, 2].

TG rickettsiae are the causative agents of epidemic typhus (*R*. *prowazekii*) and endemic typhus (*R*. *typhi*). *R*. *prowazekii* is transmitted from human to human by the human body louse while rodents are considered as the dominant natural reservoir for *R*. *typhi* and fleas serve as vectors for these bacteria. Rickettsiae primarily infect endothelial cells [3], leading to local vascular lesions and inflammatory responses that become visible as a characteristic hemorrhagic skin rash in 40–60% of the patients [1]. Symptoms of epidemic and endemic typhus are quite similar. After a 10–14 days period of latency patients suffer from high fever accompanied by headache, muscle and joint pain, nausea and vomiting. Furthermore, neurological symptoms such as confusion and stupor are common [4]. In severe cases, fatal multi-organ pathology including pneumonia, myocarditis, nephritis, hepatitis, splenomegaly and encephalitis/meningitis can occur [4, 5]. The lethality of epidemic typhus is up to 20–30% [5–7] while the course of disease of endemic typhus is usually milder. The lethality of endemic typhus is estimated to be less than 5% [7, 8] if untreated with antibiotics. Vaccines are not available.

In recent years mouse models of rickettsial infections have been established, using nearly exclusively SFG rickettsiae. While BALB/c and C57BL/6 mice are resistant to the infection with various rickettsiae, C3H/HeN mice were revealed to be susceptible [9–13]. These mice have been used in various studies to analyze immune response against rickettsiae. CD8^+^ T cells seem to be critical for protection. C3H/HeN mice depleted of CD8^+^ T cells died upon infection with a normally sublethal dose of *R*. *conorii* while CD4^+^ T cell-depleted animals showed a similar course of illness as control mice [14]. Furthermore, adoptive transfer of immune CD8^+^ T cells protected C3H/HeN mice against a lethal challenge with *R*. *conorii* [14] but also the transfer of immune CD4^+^ T cells was protective in this system [14]. The role of CD8^+^ T cells was further addressed by the infection of CD8^+^ T cell-deficient C57BL/6 MHCI^-/-^ mice and C57BL/6 Perforin^-/-^ mice that lack the cytotoxic activity of CD8^+^ T cells and NK cells with *R*. *australis*. Both animals showed enhanced lethality in this infection [15], demonstrating a critical role of CD8^+^ T cells and their cytotoxic activity in protection against SFG rickettsiae.

Important cytokines that are involved in rickettsial defense are TNFα and IFNγ. *R*. *conorii*-infected C3H/HeN mice produced enhanced serum levels of IFNγ and IL-12, the main IFNγ-inducing factor for T cells and NK cells [16], in the first days of infection [12]. Furthermore, depletion of NK cells led to reduced IFNγ release and enhanced susceptibility of C3H/HeN mice to the infection with *R*. *conorii* [12], suggesting the contribution of NK cells to early defense against rickettsiae via the release of IFNγ. Neutralization of either IFNγ or TNFα was associated with reduced nitric oxide (NO) production, led to uncontrolled bacterial growth and was fatal for C3H/HeN mice upon infection with a normally sublethal dose of *R*. *conorii* [17]. In line with these observations C57BL/6 IFNγ^-/-^ mice showed enhanced lethality upon *R*. *australis* infection compared to wild-type mice [15].

Knowledge about immune response against TG rickettsiae, however, is still rare. Depletion of NK cells enhanced the susceptibility of normally resistant C57BL/6 mice to *R*. *typhi* infection [12]. Depletion of CD8^+^ T cells as well as the neutralization of IFNγ led to enhanced bacterial growth and mortality of C3H/HeN mice in *R*. *typhi* infection [18]. We recently showed that immune CD8^+^ as well as CD4^+^ T cells are capable of protecting T and B cell-deficient C57BL/6 RAG1^-/-^ mice against *R*. *typhi* [19], a model where the bacteria persist for several months and finally cause lethal CNS inflammation [20]. These observations suggest that similar mechanisms including NK cells, T cells, IFNγ and TNFα are involved in protection against both SFG and TG rickettsiae.

The current study was performed to further clarify the protective capacity of CD4^+^ and CD8^+^ T cells and to decipher the effector mechanisms that are needed for T cell-mediated protection employing BALB/c wild-type mice and the CB17 SCID model of *R*. *typhi* infection. In CB17 SCID mice *R*. *typhi* induces splenomegaly, severe liver injury and fatal systemic inflammation [21]. Thus, the CB17 SCID model of infection reflects complications that frequently occur in patients with severe outcome of murine typhus [22]. In this model we show that the cytotoxic activity is not essential for CD8^+^ T cell-mediated protection. The release of IFNγ and other factors by CD8^+^ T cells is obviously as efficient in the control of *R*. *typhi* as direct killing of infected cells. Furthermore, we show that either CD4^+^ T_H_1 or T_H_17 cells protect against *R*. *typhi*. Here, T_H_17 cells that produce TNFα, IL-17A and IL-22 are as protective as IFNγ-releasing T_H_1 cells, provided that the non-beneficial biological effects of either TNFα or IL-17A are inhibited.

## Methods

### Ethics statement

All experimentations and procedures were approved by the Public Health Authorities (Amt fuer Gesundheit und Verbraucherschutz, Hamburg; No 88/13) and performed according to the German AnimalWelfare Act.

### Mice

BALB/c, BALB/c IFNγ^-/-^ [23], BALB/c Perforin^-/-^ [24] and congenic CB17 SCID (CB17/lcr-Prkdc^SCID^/lcrlcoCrl) mice that lack T and B cells due to a genetic autosomal recessive mutation in the Prkdc^SCID^ allele on chromosome 16 [25, 26] were bred and maintained in the animal facilities of the Bernhard Nocht Institute for Tropical Medicine, Hamburg. Animals were housed in a biosafety level 3 facility for experimentation. The facilities are registered by the Public Health Authorities (Amt für Gesundheit und Verbraucherschutz, Hamburg).

### Culture, purification and freezing of *R*. *typhi*

*R*. *typhi* (Wilmington strain) was cultivated in L929 mouse fibroblasts (ATCC CCL-1) and purified as described previously [20]. Stocks of purified bacteria were frozen in liquid nitrogen. Spot forming units (sfu) of thawed bacteria were determined by immunofocus assay as described [20].

### Purification of T cells

CD8^+^ and CD4^+^ T cells were purified employing the MagniSort Mouse CD4 and CD8 Enrichment kits from eBioscience, Frankfurt, Germany. Procedures were performed according to the manufacturer´s instructions. The purity was generally >95% as determined by flow cytometric stainings.

### Infection of mice, adoptive T cell transfer and cytokine neutralization

Mice were infected subcutaneously (s.c.) into the tail base with 2×10^6^ sfu *R*. *typhi* in 50 μl PBS. Either 1×10^6^ purified CD8^+^ or CD4^+^ T cells from naïve BALB/c, BALB/c IFNγ^-/-^ or BALB/c Perforin^-/-^ mice were adoptively transferred 1 day prior to infection. TNFα was neutralized by intraperitoneal application of 500 μg anti-TNFα (clone XT3.11, BioXCell, West Lebanon, US) in 200 μl PBS. Control mice received the same amount of isotype antibody (clone HRPN, BioXCell, West Lebanon, US). Treatment was performed every three days beginning on day 3 post infection. For the neutralization of IL-17A, anti-IL-17A (clone 13F3) was used (BioXCell, West Lebanon, US). 500 μg of the antibody were injected i.p. in 200 μl PBS every 2 days starting on day 2 post infection. Control mice received the same amount of isotype antibody (clone MOPC-21, BioXCell, West Lebanon, US).

### Clinical scoring and survival

The health status of the animals was monitored with a clinical score [20] assessing five criteria: posture (0: normal, 1: temporarily curved, 2: curved), fur condition (0: normal, 1: staring in the neck, 2: overall staring), activity (0: normal, 1: reduced, 2: strongly reduced), weight loss (0: < 10%, 1: 10–14%, 2: > 15%) and food and water uptake (0: normal, 1: reduced, 2: none). Mice were euthanized reaching a total score ≥ 8 or showing weight loss of ≥ 20%. This point in time was determined as the time of death and used to determine survival rates. The state of health of the animals was assessed by clinical scoring.

### Collection of blood samples

Blood was taken submandibular or by cardiac puncture. EDTA coated tubes (KABE Labortechnik GmbH, Nümbrecht-Elsenroth, Germany) were used for plasma samples and centrifuged at 5654×g. Serum samples were obtained by agglutination for 15–20 at RT in Eppendorf tubes followed by centrifugation for 10 min 5654×g.

### DNA preparation from purified bacteria, cell culture and organs

DNA was prepared employing the QIAamp DNA Mini Kit (Qiagen, Hilden, Germany) according to the manufacturer’s guide. 10 mg tissue was homogenized in 500 μl PBS in Precellys ceramic Kit tubes (Peqlab. Erlangen, Germany) in a Precellys 24 homogenizer (Peqlab. Erlangen, Germany) with following cycle parameters: 6000 rpm two times for 45 sec with a 60 sec break. DNA was prepared from 80 μl homogenized organs.

### Quantitative real-time PCR (qPCR)

qPCR was performed as described previously [20] by amplification of a 137 bp fragment of the *prsA* gene (RT0565) with the forward primer 5´-ACA GCT TCA AAT GGT GGG GT-3´ and reverse primer 5´-TGC CAG CCG AAA TCT GTT TTG-3´ in a standard SYBR green real-time PCR. A standard template plasmid (pCR2.1-*prsA*) was used as a reference. Reactions were performed in a Rotor Gene 6000 (Qiagen, Hilden, Germany).

### Flow cytometry

Single cell suspensions were prepared from spleen and blood. Erythrocytes were eliminated by incubating the cells in erythrocyte lysis buffer (10 mM Tris, 144 mM NH_4_Cl, pH 7.5) for 5 minutes at room temperature. Afterwards, cells were washed twice with PBS. Fc receptors were blocked with 50 μl 5% CohnII human IgG fraction (Sigma-Aldrich, Deisenhofen, Germany) in PBS or Permeabilization buffer of the FoxP3/Transcription factor staining buffer kit (eBioscience, Frankfurt, Germany). Spleen cells were restimulated with 10 ng/ml PMA and 500 ng/ml Ionomycin in 200 μl in 96well plates in the presence of 1 μl GolgiStop (BD Biosciences, Heidelberg, Germany) for 4h and permeabilized with Fixation and Permebilization buffer. For intracellular stainings antibodies were diluted in the Permeabilization buffer of the kit. The following antibodies were used at indicated dilutions: anti-mouse CD4-PE (clone GK1.5; 1:200) and CD8-PerCP-Cy5.5 (clone 53–6.7; 1:200) from BD Biosciences, Heidelberg, Germany; anti-mouse CD8-Alexa488 (clone 53–6.7; 1:200), anti-mouse CD4-FITC (clone H129.19; 1:200), anti-mouse CD8-APC (clone 53–6.7; 1:200), anti-mouse KLRG1-PE (clone 2F1/KLRG1; 1:800), anti-mouse IFNγ-PE/Dazzle (clone XMG1.2; 1:333) and anti-mouse Granzyme B-PacificBlue (clone GB11; 1:200) from Biolegend, London, UK. anti-mouse CD11a-eFluor450 (clone M17/4; 1:200) from eBioscience, Frankfurt, Germany. After staining, cells were washed and resuspended in PBS/1% PFA prior to flow cytometry. Analyses were performed with a BD Accuri C6 or BD LSR II flow cytometer (BD Biosciences, San José, USA) and FlowJo single cell analysis software (FlowJo LLC, Ashland, USA).

### Detection of cytokines in plasma and cell culture supernatants

Bead-based LEGENDplex immunoassay (BioLegend, London, UK) was used for the quantification of plasma cytokines and cytokines in cell culture supernatants. Procedures were performed according to the manufacturer’s protocol using cluster tubes (ThermoScientific, Loughborough UK). 12.5 μl of plasma from EDTA blood samples was used diluted 1:2 in assay buffer. Supernatants from bmMΦ were used non-diluted. Analyses were performed using a BD Accuri C6 (BD Biosciences, San José, USA) and LEGENDplex analysis software (BioLegend, San Diego, USA).

### Detection of serum Glutamate Pyruvate Transaminase (GPT)

Serum levels of GPT were evaluated using Reflotron GPT (ALT) stripes and Reflotron Plus device (Roche Diagnostics, Mannheim, Germany) according to the manufacturer’s instructions. Serum samples were diluted 1:3 in PBS prior to analyses.

### Generation of bmMΦ *in vitro*

Bone marrow was isolated from femur and tibia of BALB/c mice. 2×10^6^ cells were plated in petri dishes and differentiated for 12 days in IMDM (PAA, Cölbe, Germany) supplemented with 10% FCS, 2 mM L-glutamine, 5% horse serum (Biochrom, Berlin, Germany) and L929 fibroblast medium as a source of M-CSF. Medium was exchanged every 3 days. bmMΦ were harvested after 12 days and washed twice with PBS prior to use.

### Infection of bmMΦ and co-culture with T cells and recombinant cytokines *in vitro*

1×10^6^ bmMΦ were seeded into 24-well tissue culture plates and infected in duplicates with 5 *R*. *typhi* particles as determined by qPCR per cell. 2×10^6^ purified CD4^+^ T cells from either naïve BALB/c mice or immune BALB/c mice that had been infected with 2×10^6^ sfu *R*. *typhi* 7 days earlier were added to the culture after 24h. 10 μg/ml neutralizing antibodies (anti-IFNγ clone XMG1.2, anti-TNFα clone XT3.11; BioXCell, West Lebanon, US) and T cells were added simultaneously. Cells were further incubated for 48h. Alternatively, recombinant cytokines (IFNγ, 1 U/ml, TNFα, 400 U/ml; PreproTech, Hamburg, Germany) were added instead of T cells and neutralized with the same amounts of antibodies. Complete wells including supernatant were then harvested. Cells were obtained by high speed centrifugation and used for DNA preparation and qPCR detection of *R*. *typhi*. In parallel, similar cultures were performed for the quantification of nitric oxide (NO) and cytokines in the culture supernatants 24h and 48h after T cell addition.

### Detection of NO

NO was quantified by Griess reaction in supernatants of bmMΦ and co-cultures with T cells. Assays were performed in microtiter plates (Greiner Bio-One, Frickenhausen, Germany). 100 μl of sample were mixed with 50 μl Griess 1 reagent (0.5 g sulfonamide in 50 ml 1M HCl) and 50 μl Griess 2 reagent (0.15 g N-(1-Naphtyl)ethylendiamine-dihydrochloride in 50 ml H_2_O). A serial dilution of sodium nitrite (NaNO_2_) in culture medium was used as a standard (c_max_ 125 μM). The absorbance was measured at 560 nm with a Dynex MRXII spectrophotometric microplate reader (Dynex Technologies, Chantilly, USA).

### Statistical analyses

GraphPad Prism 5 software (GraphPad Software, Inc., La Jolla, USA) was used for statistical analysis. The proportion of surviving animals was analyzed with Log-rank (Mantel-Cox) test. For comparison between multiple groups One-way ANOVA (Kruskal Wallis test followed by Dunn´s post test) was used.

## Results

### BALB/c wild-type mice generate a persistent cytotoxic CD8^+^ T cell and CD4^+^ T_H_1 response upon *R*. *typhi* infection

**Fig 1:**
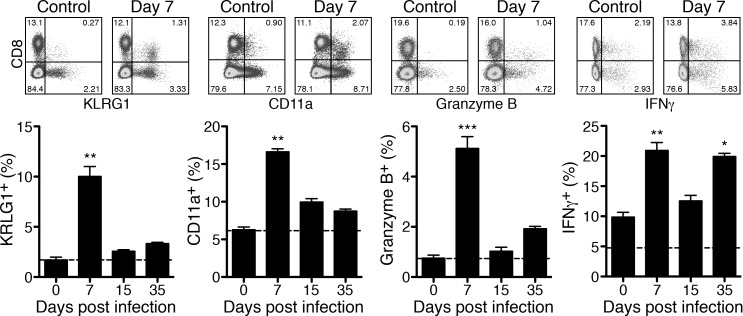
BALB/c mice generate cytotoxic CD8^+^ cells that are sporadically reactivated. BALB/c mice were infected with 1×10^6^ sfu *R*. *typhi*. Control mice received PBS instead and were used as "day 0" control. Spleen cells were isolated and stained for CD8, KLRG1 and CD11a or restimulated with PMA/Ionomycin for 4h and stained for CD8 and intracellular IFNγ and Granzyme B. The dot plots show example stainings from day 7 post infection. Mice were analyzed for cytokine and Granzyme B expression on day 0, 7 and 15 (n = 6) and day 35 (n = 4). 3–4 mice were analyzed for KLRG1 and CD11a expression. Graphs show the percentage of KLRG1^+^, CD11a^+^, Granzyme B^+^ and IFNγ^+^ T cells among CD8^+^ T cells (y-axis) at indicated days post infection (x-axis). Graphs show combined results from 2 independent experiments. Statistical analysis was performed by One-way ANOVA (Kruskal Wallis test followed by Dunn´s post test). Asterisks indicate significant differences compared to day 0 (*p<0.05, **p<0.01, ***p<0.001).

BALB/c mice do not develop symptomatic disease upon *R*. *typhi* infection but the bacteria persist [20]. Control of the bacteria in these mice is mediated by the adaptive immune response because T and B cell deficient congenic mice are highly susceptible and succumb to the infection within three weeks [21]. Therefore, we first analyzed the T cell response in BALB/c mice upon *R*. *typhi* infection. For this purpose, spleen cells from *R*. *typhi*-infected BALB/c mice were isolated during the course of infection at day 7, 15 and 35. Control mice were treated with PBS (day 0). Cells were restimulated with PMA and ionomycin for the intracellular staining of effector molecules (Granzyme B and IFNγ). Additionally, surface CD8, CD4, CD11a and KLRG1 (inhibitory killer cell lectin-like receptor G1) were stained. CD11a and KLRG1 are upregulated during immune response, allowing to distinguish CD11a^+^ antigen-experienced CD8^+^ T cells [27] and KLRG1^+^ activated effector CD8^+^ T cells from naïve T cells that are negative for these markers [28, 29]. Cells were analyzed by flow cytometry and gated either on CD8^+^ or CD4^+^ T cells. Total numbers of CD8^+^ T cells did not significantly increase at any point in time but a significant increase of the frequency of CD11a^+^ and KLRG1^+^ CD8^+^ T cells was observed on day 7 after infection (Fig 1). At this point in time 16.63±0.39% of the CD8^+^ T cells expressed CD11a and 10.02±0.98% were positive for KLRG1 while 6.27±0.37% CD11a^+^ and 1.69±0.29% KLRG1^+^ CD8^+^ T cells were detectable in control animals (day 0). In addition, the percentage of CD8^+^ T cells that expressed Granzyme B and IFNγ was significantly enhanced on day 7. 5.12±0.47% of the CD8^+^ T cells were Granzyme B^+^ and 20.93±1.31% CD8^+^ T cells expressed IFNγ compared to 0.75±0.28% Granzyme B^+^ and 9.87±0.78% IFNγ^+^ cells in control mice (day 0). The CD8^+^ T cell response declined until day 15 but did not reach background levels again. On the contrary, CD8^+^ T cells appeared to be reactivated on day 35 post infection again. At this late point in time IFNγ expression was significantly enhanced and IFNγ-producing CD8^+^ T cells reached frequencies (19.93±0.52%) comparable to those observed on day 7 post infection. A similar trend was true for the expression of Granzyme B (Fig 1).

**Fig 2:**
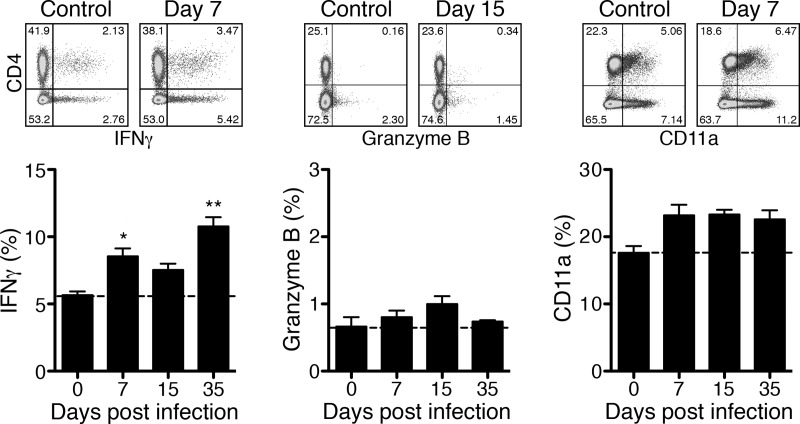
BALB/c mice generate CD4^+^ T_H_1 cells that are sporadically reactivated. Spleen cells from the same mice as described in Fig 1 were stained for CD4, CD11a and for intracellular IFNγ and Granzyme B after PMA/Ionomycin restimulation. The dot plots show example stainings from day 7 or day 15 post infection. Graphs show the percentage of CD11a^+^, IFNγ^+^ and Granzyme B^+^ T cells among CD4^+^ T cells (y-axis) at indicated days post infection (x-axis). Statistical analysis was performed by One-way ANOVA (Kruskal Wallis test followed by Dunn´s post test). Asterisks indicate significant differences compared to day 0 (*p<0.05, **p<0.01, ***p<0.001).

As seen for CD8^+^ T cells the absolute cell counts of CD4^+^ T cells did not increase during the course of infection but the frequency of CD4^+^ T cells that expressed intracellular IFNγ was significantly increased on day 7 post infection (8.54±0.60% compared to 5.66±0.27% in control mice (day 0)). Similar to activated CD8^+^ T cells, IFNγ-expressing CD4^+^ T cells declined until day 15 and increased again on day 35 reaching even higher frequencies (10.78±0.68%) compared to day 7 (Fig 2, left). This indicates sporadic reactivation also of CD4^+^ T cells during chronic infection.

In persistent infections CD4^+^ T cells can acquire cytotoxic function. These cells up-regulate CD11a and express cytolytic mediators including Granzyme B [30]. Granzyme B was not expressed at all by CD4^+^ T cells during *R*. *typhi* infection (Fig 2, middle).

Approximately 17% of the CD4^+^ T cells were positive for CD11a in naïve mice (Fig 2, right). CD11a was not expressed at significantly enhanced levels by CD4^+^ T cells in *R*. *typhi*-infected BALB/c mice at any point in time (Fig 2). Thus, CD4^+^ T cells in *R*. *typhi*-infected animals do not show characteristics of cytotoxic cells. These findings demonstrate that BALB/c mice mount a cytotoxic CD8^+^ T cell effector response and generate IFNγ-expressing CD4^+^ T_H_1 effector cells both of which become reactivated in persistent infection with similar kinetics.

### Adoptive transfer of CD8^+^ and CD4^+^ T cells protects CB17 SCID mice against *R*. *typhi* infection

**Fig 3:**
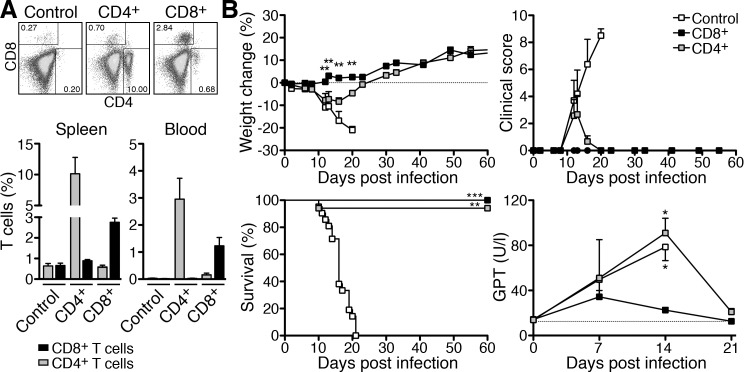
Adoptive transfer of either CD8^+^ or CD4^+^ T cells protects CB17 SCID mice from severe disease and death. CD4^+^ and CD8^+^ T cells were isolated from naïve BALB/c mice. 1×10^6^ T cells were adoptively transferred into CB17 SCID mice 1 day prior to infection with 1×10^6^ sfu *R*. *typhi* or treatment with PBS (Control). Spleen and blood of the animals were analyzed for the presence of T cells on day 7 post infection. The dot plots show example stainings of spleen cells for CD4 and CD8 from a CB17 SCID control mouse, a CD4^+^ T cell recipient (middle) and a CD8^+^ T cell recipient (right). The graphs show the statistical analysis of the spleen (left) and blood (right). The percentage (y-axis) of CD4^+^ and CD8^+^ T cells (x-axis) was determined for control mice (n = 5; white bars), CD4^+^ T cell recipients (n = 5; gray bars) and CD8^+^ T cell recipients (n = 5; black bars). On average CD4^+^ T cell recipients contained 10.1±2.6% CD4^+^ T cells while CD8^+^ T cells were virtually absent (0.9±0.1%). 2.8±0.2% CD8^+^ T cells were detected in CD8^+^ T cells while CD4^+^ T cells were absent (0.6±0.1%). The percentage of T cells in the blood was lower (3.0±0.8% CD4^+^ T cells in CD4^+^ T cell recipient and 1.2±0.3% CD8^+^ T cells in CD8^+^ recipients) (**A**). Weight change (n = 5 for control animals and n = 6 for T cell recipient groups), clinical score (n = 5 for control animals and n = 6 for T cell recipient groups), survival (n = 11 for each group) and serum GPT levels (n = 3–5 for each group) were assessed (y-axis) at indicated points in time (x-axis). Differences in the weight change between CD4^+^ and CD8^+^ T cell recipients were compared by Mann-Whitney U test at indicated points in time. Statistical analysis of GPT levels was performed by One-way ANOVA (Kruskal Wallis test followed by Dunn´s post) test. Asterisks indicate significant differences compared to day 0 (*p<0.05, **p<0.01). The survival graph shows combined results from 2 independent experiments. Statistical analysis was performed with Log-rank (Mantel-Cox) test. Asterisks indicate significant differences compared to control animals (**p<0.01, ***p<0.001) (**B**).

We next addressed the question whether either CD8^+^ or CD4^+^ T cells can protect mice against *R*. *typhi* infection. For this purpose, we performed adoptive transfer of T cells from BALB/c mice into susceptible congenic T and B cell-deficient CB17 SCID mice that were infected with *R*. *typhi*. Either CD4^+^ or CD8^+^ T cells were isolated from naïve BALB/c mice. T cells were then adoptively transferred into CB17 SCID mice one day prior to *R*. *typhi* infection. Control mice received PBS instead of T cells and were infected with *R*. *typhi*. The presence of CD4^+^ and CD8^+^ T cells was analyzed in spleen and blood on day 7 post infection in all groups. As expected, neither CD4^+^ T cells (spleen: 0.64±0.12%; blood: 0.03±0.01%) nor CD8^+^ T cells (spleen: 0.66±0.11%; blood: 0.02±0.00%) were detectable in CB17 SCID control mice that did not receive T cells (Fig 3A).

In CD4^+^ T cell recipients 10.13±2.65% of the spleen cells were CD4^+^ while CD8^+^ T cells were clearly absent (0.89±0.06%). The same was true for the blood. CD4^+^ T cells constituted 2.96±0.77% of the leukocytes in the blood while CD8^+^ T cells were not detectable (0.03±0.01%). Vice versa CD8^+^ T cells were present in the spleen of CD8^+^ T cell recipients (2.75±0.21%) and in the blood of these animals (1.23±0.31%) while CD4^+^ T cells were absent (spleen: 0.58±0.09%; blood: 0.16±0.06%) (Fig 3A).

The health status of the animals was monitored by measuring body weight and evaluation by a clinical score. Weight change, clinical score and survival rates of CD4^+^ and CD8^+^ recipients and control mice are depicted in Fig 3B. *R*. *typhi*-infected CB17 SCID control mice continuously lost weight beginning around day 10 and developed a high clinical score >8 and severe disease until day 20. Mice showing a score ≥8 were euthanized. This point in time was defined as time of death to analyze survival rates. All CB17 SCID control mice succumbed to the infection before day 21. In contrast, CD8^+^ T cell recipients neither lost weight nor showed signs of disease at any point in time and all animals survived the infection (Fig 3B). *R*. *typhi*-infected CB17 SCID mice that received CD4^+^ T cells showed temporary weight loss with similar kinetics as control animals which was significant compared to CD8^+^ T cell recipients and a low clinical score with a maximum of 4 around day 12 and 13 (*p* = 0.06 compared to CD8^+^ T cell recipients) (Fig 3B). Surprisingly, except for one animal CD4^+^ T cell recipient mice recovered until day 20 and survived the infection (Fig 3B). In addition, GPT levels were detected in the serum during the course of infection as a measure for liver damage that is observed in CB17 SCID mice upon *R*. *typhi* infection [21]. In concordance with temporary disease, GPT levels significantly increased in CD4^+^ T cell recipients until day 14 post infection, reaching comparable levels as in infected control mice. GPT concentrations then declined to background levels again until day 21 when the mice recovered. Serum GPT levels were not increased in CD8^+^ T cell recipients at any point in time (Fig 3B). These data show that both CD8^+^ as well as CD4^+^ T cells are capable of protecting CB17 SCID mice from death upon *R*. *typhi* infection although CD4^+^ T cells are less efficient in protection from *R*. *typhi*-induced disease.

### Serum levels of IFNγ and TNFα and bacterial load in CD4^+^ and CD8^+^ T cell recipients

**Fig 4:**
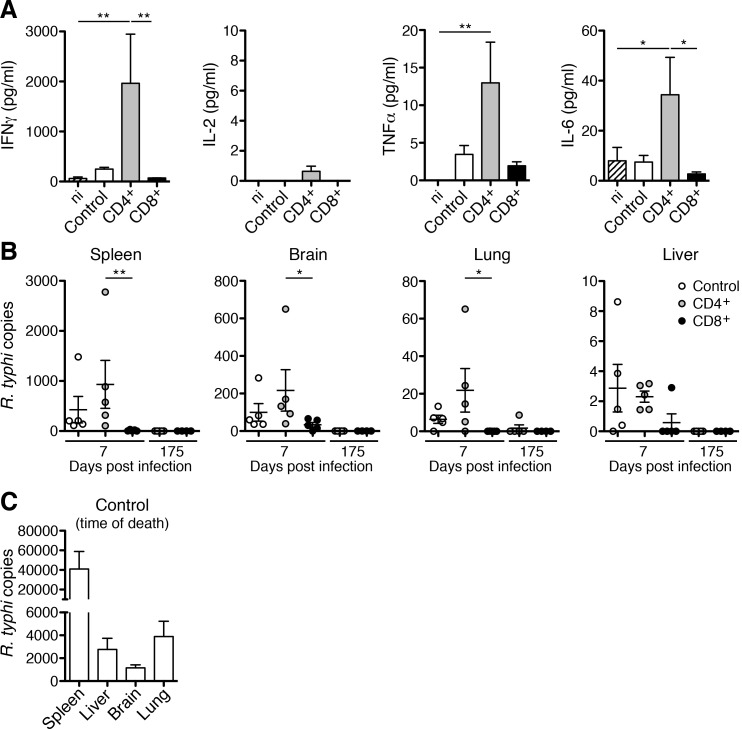
Adoptively transferred CD4^+^ T cells produce IFNγ and TNFα and both CD4^+^ and CD8^+^ T cells provide long-term control of *R*. *typhi in vivo*. From the same mice as described in Fig 3 cytokine levels (y-axis) were determined in plasma on day 7 post infection (n = 7 for each group as indicated on the x-axis). In addition, plasma from non-infected PBS-treated mice (ni; n = 6) was analyzed. Statistical analysis was performed by One-way ANOVA (Kruskal Wallis test followed by Dunn´s post test). Asterisks indicate significant differences (*p<0.05, **p<0.01) (**A**). Bacterial content (y-axis) in the organs indicated above was quantified by qPCR in each group (*R*. *typhi*-infected control mice: open circles, CD4^+^ T cell recipients: gray circles; CD8^+^ T cell recipients: black circles) on day 7 (n = 5 for each group) and when the experiment was terminated on day 175 (n = 5 for control mice and CD4^+^ T cell recipients; n = 4 for CD8^+^ T cell recipients) post infection (x-axis). Each symbol represents a single mouse. Statistical analysis was performed by One-way ANOVA (Kruskal Wallis test followed by Dunn´s post test). Asterisks indicate significant differences (*p<0.05, **p<0.01) (**B**). Bacterial content was quantified in *R*. *typhi*-infected control animals (n = 5) at the time of death in the indicated organs (x-axis) (**C**).

As an indicator for the activation of adoptively transferred T cells we further analyzed serum cytokine levels on day 7 post infection in control mice, CD4^+^ and CD8^+^ T cell recipients. Non-infected CB17 SCID mice that received PBS were used as an additional control. At this early point in time in infection, enhanced amounts of IFNγ (248.80±34.54 pg/ml) and TNFα (3.47±1.17 pg/ml) were already present in the serum of *R*. *typhi*-infected control mice compared to non-infected animals where TNFα was not detectable at all and IFNγ was present at background levels (60.13±30.12 pg/ml). IL-6 was not yet enhanced in *R*. *typhi*-infected CB17 SCID mice (7.48±2.64 pg/ml) compared to non-infected animals (4.90±4.90 pg/ml) and IL-2 was not detectable in these mice as expected. IL-2 was also not measurable in CD8^+^ T cell recipients. TNFα (1.94±0.54 pg/ml) was slightly enhanced compared to non-infected animals and comparable to levels measured in infected control animals (3.47±1.17 pg/ml). IFNγ (70.06±23.54 pg/ml) and IL-6 (2.74±0.79 pg/ml) were detected at background levels in CD8^+^ T cell recipients, indicating that the immune response is already being terminated. In contrast to CD8^+^ T cell recipients, IFNγ concentrations were strongly and significantly enhanced in the sera of animals that had received CD4^+^ T cells (1963±982.30 pg/ml). Similar was also true for TNFα (12.97±5.42 pg/ml) and IL-6 (34.36±14.95 pg/ml) although levels of these cytokines were still quite low. In addition, very low amounts of IL-2 were measurable in the sera from CD4^+^ T cell recipients (0.63±0.33 pg/ml) (Fig 4A).

Other T cell-derived cytokines including IL-17A/F and IL-22 were generally not detectable. These data show that adoptively transferred CD4^+^ T cells produce IFNγ and enhance the inflammatory response at day 7 post infection while the immune response seems to be already terminated in CD8^+^ T cell recipients at this time.

To analyze the capacity of both CD4^+^ and CD8^+^ T cells to eliminate the bacteria *in vivo* we further determined the bacterial load in different organs of all groups of mice on day 7 post infection by qPCR. Highest numbers of bacteria were generally detectable in the spleen followed by the brain, lung and liver in CB17 SCID mice (Fig 4B and [21]). At this early point in time after *R*. *typhi* infection bacterial numbers were still low. In *R*. *typhi*-infected control mice 427.30±263.80 copies were detectable in the spleen, 99.57±46.64 copies in the brain, 6.40±2.14 copies in the lung and 1.43±0.87 copies in the liver. The bacterial load in these organs was unaltered in CD4^+^ T cell recipients (spleen: 471.20±168.1 copies, brain: 108.40±27.93 copies, lung: 11.10±5.37 copies, liver: 2.09±0.39 copies) at this point in time but significantly reduced in animals that received CD8^+^ T cells. In fact, the bacteria were already almost absent in the organs of this group of mice (spleen: 11.23±6.26 copies, lung: 0.03±0.01 copies, liver: 0.58±057 copies) except for the brain (33.55±12.41 copies) (Fig 4B). In control animals, bacterial numbers further increased until death (spleen: 40931±17971 copies, brain: 1151±269.9 copies, lung: 3897±1334 copies, liver 2767±970.4 copies) (Fig 4C).

Five mice of the CD4^+^ and CD8^+^ recipient groups were further monitored for 175 days. One mouse of the CD8^+^ recipient group died on day 85 for undefined reasons. This animal did not show typical symptoms of *R*. *typhi*-induced disease and was not further analyzed. All other animals survived. The bacteria were not detectable in the organs from the CD8^+^ T cell recipients at this very late point in time (Fig 4B). Furthermore, the bacteria were also absent in the organs from *R*. *typhi*-infected CD4^+^ recipient mice except for one animal that showed very low numbers of bacteria in the lung (8.65 copies; Fig 4B). This mouse, however, did not show any signs of disease. These results demonstrate that adoptively transferred CD8^+^ as well as CD4^+^ T cells are capable of eliminating *R*. *typhi* and provide long-term control of the bacteria.

### Role of IFNγ and Perforin for CD4^+^ and CD8^+^ T cell-mediated protection of CB17 SCID mice against *R*. *typhi*

**Fig 5:**
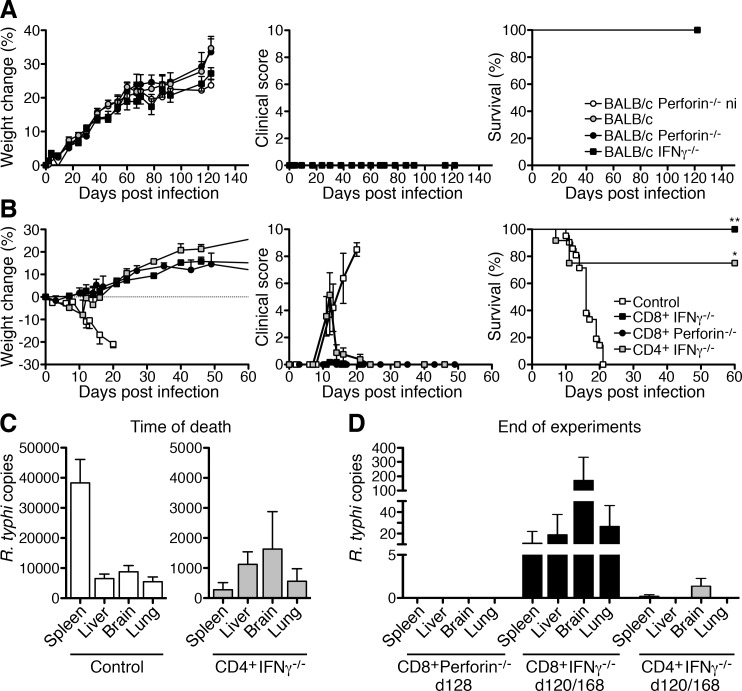
CD8^+^Perforin^-/-^, CD8^+^ IFNγ^-/-^ and CD4^+^IFNγ^-/-^ T cells are still protective. BALB/c wild-type mice (gray circles; n = 5), BALB/c Perforin^-/-^ (black circles; n = 5) and BALB/c IFNγ^-/-^ mice (black squares; n = 5) were infected with 1×10^6^ sfu *R*. *typhi*. Non-infected BALB/c Perforin^-/-^ mice (white circles; n = 5) were used as a control. Weight change (left), clinical score (middle) and survival (y-axis) was assessed at indicated points in time (x-axis) (**A**). CD8^+^ and CD4^+^ T cells were isolated from BALB/c Perforin^-/-^ and IFNγ^-/-^ mice. 1×10^6^ CD8^+^ IFNγ^-/-^ (black squares), CD8^+^ Perforin^-/-^ (black circles) and CD4^+^ IFNγ^-/-^ T cells (gray squares) were adoptively transferred into CB17 SCID mice 1 day prior to the infection with 1×10^6^ sfu *R*. *typhi*. Control animals were treated with PBS instead receiving T cells (white squares). Weight change (left; n = 5 for each group), clinical score (middle; n = 5 for each group) and survival (right; n = 5 for CD8^+^ Perforin^-/-^ recipients and n = 10–11 for CD8^+^ and CD4^+^ IFNγ^-/-^ recipients) was assessed (y-axis) at indicated points in time (x-axis). The survival graph shows combined results from 2 independent experiments. Statistical analysis was performed with Log-rank (Mantel-Cox) test. Asterisks indicate significant differences compared to control animals (*p<0.05, **p<0.01) (**B**). All *R*. *typhi*-infected CB17 SCID control animals and two mice of the CD4^+^ T IFNγ^-/-^ cell recipient group died until day 20. At the time of death, the bacterial load (y-axis) was determined in the indicated organs (x-axis) in these animals (**C**). The bacterial load (y-axis) was also determined in the organs of surviving animals. Organs were taken at indicated points in time from CD8^+^Perforin^-/-^ (day 128 post infection; n = 4), CD8^+^ IFNγ^-/-^ (n = 6) and CD4^+^IFNγ^-/-^ T cell recipients (n = 8). For the transfer of CD8^+^ IFNγ^-/-^ and CD4^+^ IFNγ^-/-^ T cells the results from two independent experiments that were terminated on day 120 and day 168 post infection are shown (x-axis). The bacteria were not detectable at all in CD8^+^Perforin^-/-^ T cell recipients while low amounts of *R*. *typhi* were present in five animals of the CD8^+^ IFNγ^-/-^ and two mice of the CD4^+^IFNγ^-/-^ T cell recipient groups. The figure shows the bacterial content in the organs of all animals (mean±SEM) (**D**).

The results presented so far show that both CD4^+^ and CD8^+^ T cells can mediate protection against *R*. *typhi* in this model of infection. We next asked which effector functions are required by both cell types to mediate protection and focused on IFNγ as an activator of bacterial killing by macrophages [31, 32] and Perforin which is critical for cell-mediated cytotoxicity [33–35]. We first infected BALB/c mice lacking either IFNγ or Perforin with *R*. *typhi*. Neither BALB/c IFNγ^-/-^ nor BALB/c Perforin^-/-^ mice showed symptoms of disease at any point in time. All animals survived the infection for more than 120 days (Fig 5A), demonstrating long-term protection in the absence of one or the other molecule.

To further elucidate the requirement of IFNγ and Perforin for CD4^+^ and CD8^+^ T cell-mediated protection we adoptively transferred either purified CD4^+^ or CD8^+^ T cells from BALB/c IFNγ^-/-^ or Perforin^-/-^ mice into CB17 SCID mice. Mice were infected with *R*. *typhi* one day after T cell transfer. *R*. *typhi*-infected CB17 SCID mice that had received CD4^+^ IFNγ^-/-^ T cells developed a clinical score and lost body weight between day 9 and 14. However, 70% of these animals recovered and survived the infection (Fig 5B). *R*. *typhi*-infected CB17 SCID mice that had either received CD8^+^ T cells from IFNγ^-/-^ or Perforin^-/-^ mice never showed any signs of disease and all of the mice survived the infection (Fig 5B).

At the time of death *R*. *typhi*-infected control animals had high bacterial loads in all organs (spleen: 38333±7786 copies; liver: 6507±1529 copies; brain: 8781±2093 copies; lung: 5420±1620 copies) as observed before while strongly reduced numbers of bacteria were detectable in the organs of the two mice of the CD4^+^ T cell recipient group that succumbed to the infection (spleen: 277.5±237.6 copies; liver: 1123±416.8 copies; brain: 1632±1243 copies; lung: 561.8±414.8 copies) (Fig 5C). Surviving animals were further followed for more than 120 days and did never show symptoms of disease again. Organs were analyzed for bacterial content when the experiments were terminated (between day 120 and 168 p.i.). Bacterial copy numbers of individual animals are given in table 1 and Fig 5D shows the statistical analysis. *R*. *typhi* was not detectable at all in the organs of CD8^+^Perforin^-/-^ T cell recipients (Fig 5D and Table 1). However, low numbers of bacteria were detectable in the majority of the CD8^+^IFNγ^-/-^ T cell recipients (5 out of 6 mice). Here, *R*. *typhi* was predominantly found in the brain (206.0±192.8 copies) while the lung, liver and spleen were much less affected (Fig 5D and Table 1). In contrast, only 2 out of 8 mice of the CD4^+^IFNγ^-/-^ T cell recipients showed low numbers of bacteria in the brain and 1 mouse was positive in the spleen (Fig 5D and Table 1).

**Table 1:** Bacterial content in the organs of surviving CD8^+^Perforin^-/-^, CD8^+^IFNγ^-/-^ and CD4^+^IFNγ^-/-^ T cell recipients.

Transferred T cells	Mouse #	Spleen	Liver	Brain	Lung
**CD8**^**+**^**IFNγ**^**-/-**^	1	ND	ND	ND	ND
	2	ND	ND	**13.53**	**5.10**
	3	**65.65**	**112.70**	**17.95**	**119.50**
	4	ND	ND	**977.00**	ND
	5	ND	ND	**6.62**	**34.30**
	6	ND	ND	**15.00**	ND
**CD8**^**+**^**Perforin**^**-/-**^	1	ND	ND	ND	ND
	2	ND	ND	ND	ND
	3	ND	ND	ND	ND
	4	ND	ND	ND	ND
**CD4**^**+**^**IFNγ**^**-/-**^	1	ND	ND	ND	ND
	2	ND	ND	ND	ND
	3	ND	ND	ND	ND
	4	**1.39**	ND	**6.00**	ND
	5	ND	ND	**4.78**	ND
	6	ND	ND	ND	ND
	7	ND	ND	ND	ND
	8	ND	ND	ND	ND

Organs were analyzed by *prsA*-specific qPCR at the end of the experiment (CD8^+^Perforin^-/-^: day 128, CD8^+^IFN**γ**^-/-^ and CD4^+^IFN**γ**^-/-^ recipients days 120 and 168 p.i.). The table shows the actually measured bacterial copy numbers in individual animals from the experiments shown in Fig 5. ND: not detectable

These results demonstrate that CD8^+^ T cells that either lack cytotoxic function or IFNγ can control the bacteria for a long period of time. For this control, IFNγ seems to be even more critical than the cytotoxic activity. On the other hand, IFNγ-deficient CD4^+^ T cells are protective and as efficient in long-term bacterial control as wild-type CD4^+^ T cells, suggesting that IFNγ is dispensable for CD4^+^ T cell-mediated protection.

### CD4^+^ T cells activate the bactericidal activity of macrophages via IFNγ and TNFα *in vitro*

**Fig 6:**
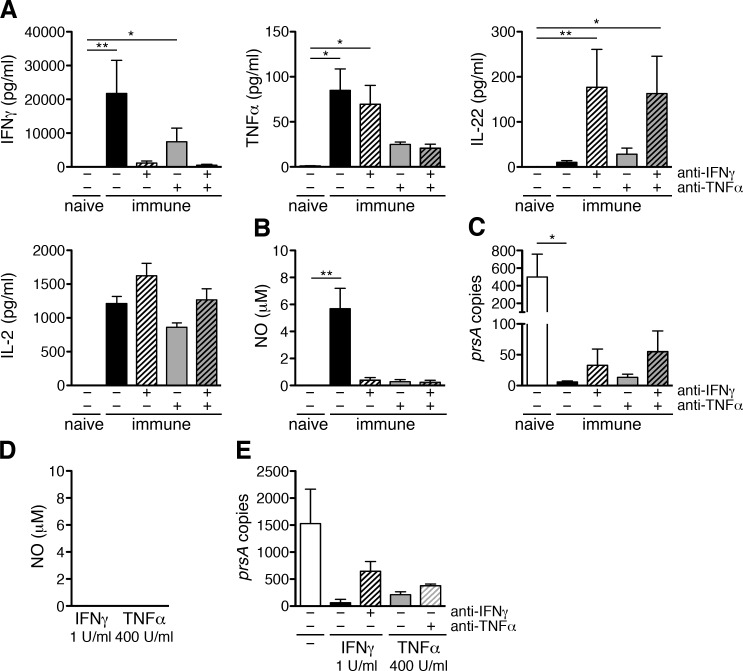
Immune CD4^+^ T cells induce NO release by *R*. *typhi*-infected macrophages *in vitro* and inhibit bacterial growth via IFNγ and TNFα. 1×10^6^ bone-marrow-derived BALB/c macrophages were infected with 5 copies of *R*. *typhi* per cell one day prior to the addition of 2×10^6^ purified CD4^+^ T cells from either naïve or immune BALB/c mice (day 7 post infection). IFNγ and TNFα were neutralized by the addition of 10 μg/ml anti-IFNγ and/or anti-TNFα as indicated on the x-axis. Cytokines were quantified in the supernatants 72h after T cell addition by LEGENDplex assay. IFNγ (left, y-axis), TNFα (middle, y-axis), IL-22 (right, y-axis) and IL-2 (below, left) are shown. Other cytokines were not detectable (**A**). In addition, NO was detected 72h after T cell addition (**B**). Bacterial content in the cultures (y-axis) was assessed by *prsA*-specific qPCR 72h after T cell addition (**C**). 1×10^6^ bone-marrow-derived BALB/c macrophages were treated with recombinant IFNγ (1 U/ml) or TNFα (400 U/ml). NO was quantified in the cell culture supernatants after 72h (**D**). 1×10^6^ bone-marrow-derived BALB/c macrophages were infected with 5 copies of *R*. *typhi* per cell one day prior to the addition of recombinant IFNγ (1 U/ml) or TNFα (400 U/ml). The cytokines were neutralized by simultaneous addition of either anti-TNFα or anti-IFNγ (10 μg/ml each) as indicated on the x-axis. Bacterial content in the cultures (y-axis) was assessed by *prsA*-specific qPCR 72h after cytokine addition (**E**). Graphs show the mean±SEM of combined results from 2 independent experiments (n = 4 T cells from each group of mice (A-C) and n = 2 for the treatment with recombinant cytokines (D-E)). Statistical analysis was performed by One-way ANOVA (Kruskal-Wallis test followed by Dunn´s post test). Asterisks indicate significant differences (*p<0.05, **p<0.01).

The observation that still 70% of *R*. *typhi*-infected CB17 SCID mice that received IFNγ^-/-^ CD4^+^ T cells survived the infection was surprising. Another cytokine that can activate phagocytes for bacterial killing and may compensate for the absence of IFNγ is TNFα [36] which was produced by adoptively transferred CD4^+^ T cells in *R*. *typhi*-infected CB17 SCID mice (Fig 4A). To clarify the contribution of TNFα to CD4^+^ T cell-mediated bacterial killing, we analyzed the impact of CD4^+^ T cell-derived TNFα and IFNγ on the activation of *R*. *typhi*-infected macrophages. For this purpose, we isolated CD4^+^ T cells from either naïve or *R*. *typhi*-infected BALB/c wild-type mice on day 7 post infection and incubated the cells with *R*. *typhi*-infected macrophages. TNFα and IFNγ were inhibited by the addition of neutralizing antibodies. Cytokines and NO were quantified in the supernatants and bacterial growth was assessed by qPCR. Fig 6A shows that CD4^+^ T cells from naïve mice did not react to *R*. *typhi*-infected macrophages at all with cytokine production while cultures containing immune T cells from *R*. *typhi*-infected animals produced high amounts of IFNγ (21749±9799 pg/ml) in addition to TNFα (84.97±23.68 pg/ml) (Fig 6A). The release of IFNγ was strongly but not completely inhibited by the addition of anti-IFNγ (1141±607 pg/ml) and also to some extent upon neutralization of TNFα (6407±3047 pg/ml). Correspondingly, IFNγ release was further inhibited by a combination of anti-IFNγ and anti-TNFα (520.8±270.3 pg/ml) (Fig 6A, left).

TNFα was reduced in the presence of anti-TNFα (24.93±2.68 pg/ml) and a combination of both antibodies (20.70±4.41 pg/ml) but not influenced by the addition of anti-IFNγ (69.56±20.93 pg/ml) (Fig 6A, middle). Although neither the neutralization of IFNγ nor TNFα was complete, the release of IFNγ and TNFα was not significantly enhanced anymore compared to cultures containing naïve T cells. Neutralization of IFNγ further led to the release of the T_H_17 cytokine IL-22 by immune CD4^+^ T cells (176.90±84.08 pg/ml) which was hardly detectable in the absence of anti-IFNγ (10.37±3.65 pg/ml) and comparably increased upon neutralization of both IFNγ and TNFα (162.90±82.76 pg/ml) (Fig 6A, right). IL-2 was not significantly altered in the presence of the antibodies (Fig 6A, below left) and other cytokines were not detectable. Because T cells are major cellular sources of IFNγ and IL-22 [37, 38], we conclude that these cytokines are produced by the T cells upon antigen recognition rather than by the macrophages and similar is likely true for TNFα in this system.

We further assessed the activation of the bactericidal function of *R*. *typhi*-infected macrophages by measuring nitric oxide (NO). Immune CD4^+^ T cells from wild-type mice induced the release of significant amounts of NO (5.69±1.51 μM) by *R*. *typhi*-infected macrophages (Fig 6B). Although cytokine neutralization was incomplete (Fig 6A), NO release was comparably and nearly completely abrogated in the presence of either anti-IFNγ (0.40±0.19 μM), anti-TNFα (0.28±0.16 μM) or a combination of both antibodies (0.24±0.15 μM) (Fig 6B). Moreover, immune but not naïve BALB/c CD4^+^ T cells strongly inhibited bacterial growth in infected macrophages *in vitro* (Fig 6C). Compared to cultures containing naïve CD4^+^ T cells where 267.6±57.92 *R*. *typhi prsA* gene copies were detected, the bacterial content was significantly reduced to 4.86±2.18 copies in the presence of immune CD4^+^ T cells (Fig 6C). Bacterial growth was partially restored in the presence of anti-IFNγ (32.35±26.62 *prsA* copies) and a combination of anti-IFNγ and anti-TNFα (54.05±34.12 *prsA* copies) while the effect of anti-TNFα alone on bacterial growth was less pronounced (10.73±5.86 *prsA* copies) (Fig 6C). Bacterial growth, however, was not completely restored by the addition of neutralizing antibodies and differences were not statistically significant compared to untreated cultures which may be ascribed to incomplete cytokine neutralization (Fig 6A).

To further elucidate a direct influence of IFNγ and TNFα on bacterial growth, additional cultures of *R*. *typhi*-infected macrophages were treated with recombinant IFNγ and TNFα in the absence of T cells. Neither IFNγ nor TNFα induced the release of detectable levels of cytokines or NO (Fig 6D) but clearly inhibited bacterial growth. In the absence of recombinant cytokines 1518±367.4 *R*. *typhi prsA* gene copies were detectable while only 90.35±40.16 *prsA* copies and 63.49±31.87 *prsA* copies were measured in the presence of recombinant TNFα or IFNγ, respectively. This effect was partially abolished upon neutralization of the respective cytokine (TNFα+anti-TNFα: 412.3±42.02 *prsA* copies, IFNγ+anti-IFNγ: 400.8±73.55 *prsA* copies). This experiment was performed twice so that statistical analysis was not performed (Fig 6E). These data demonstrate that both T cell-derived IFNγ and TNFα can contribute to the activation of macrophage bactericidal activity and bacterial killing by CD4^+^ T cells.

### The neutralization of TNFα in *R*. *typhi*-infected CD4^+^IFNγ^-/-^ T cell recipients mitigates disease and leads to enhanced survival

**Fig 7:**
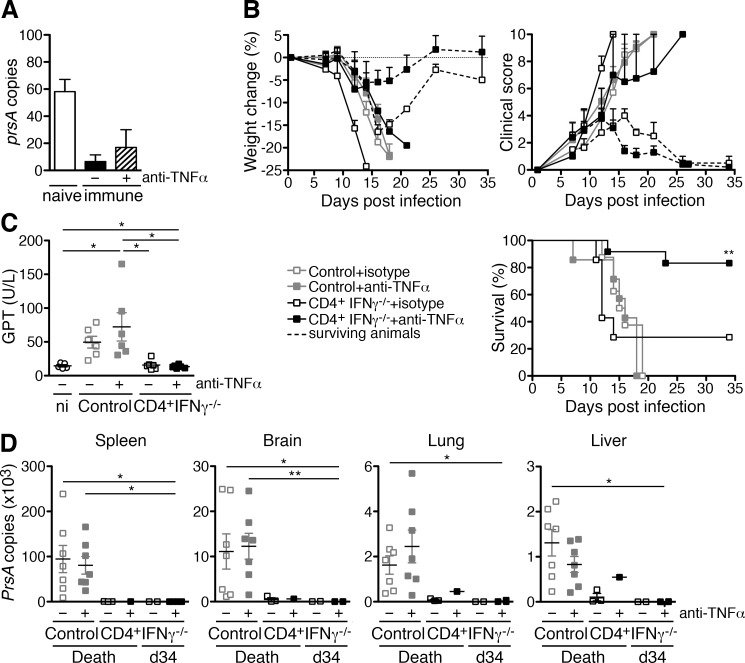
Enhanced protection by CD4^+^IFNγ^-/-^ T cells in the absence of TNFα. 1×10^6^ bone-marrow-derived BALB/c macrophages were infected with 5 copies of *R*. *typhi* per cell one day prior to the addition of 2×10^6^ purified CD4^+^ T cells from either naïve or immune BALB/c IFNγ^-/-^ mice (day 7 post infection). TNFα was neutralized by simultaneous addition of 10 μg/ml anti-TNFα. Bacterial content in the cultures (y-axis) was assessed by *prsA*-specific qPCR 72h after T cell addition. Graphs show the mean and SEM of combined results from two independent experiments (n = 4 T cells from each group of mice) (**A**). CB17 SCID mice (n = 7 for each group) were infected with 1×10^6^ sfu *R*. *typhi*. 1×10^6^ purified CD4^+^ T cells from BALB/c IFNγ^-/-^ mice were adoptively transferred one day prior to the infection with *R*. *typhi*. Control groups of mice received PBS instead. TNFα was neutralized by intraperitoneal application of 500 μg anti-TNFα every three days beginning on day 3 post infection. Control animals received equal amounts of isotype antibody. The state of health of the mice was monitored by weight change (y-axis, upper left) and a clinical score (y-axis, upper right) and the survival rates (y-axis, below) were assessed. Dotted lines show the data for surviving animals of the isotype- and anti-TNFα-treated groups of CD4^+^IFNγ^-/-^ recipients. Statistical analysis of survival rates was performed with Log-rank (Mantel-Cox) test. Asterisks indicate significant differences compared to control animals (**p<0.01) (**B**). Serum GPT levels (y-axis) were assessed from all groups of animals at the time of death and in surviving animals at the end of the experiment (day 34). Combined results are shown. Each dot represents a single mouse. Statistical analysis was performed by One-way ANOVA (Kruskal Wallis test followed by Dunn´s post test) (*p<0.05) (**C**). The bacterial content (y-axis) in the organs was quantified by *prsA*-specific qPCR from all animals that succumbed to the infection at the time of death and from surviving animals at the end of the experiment (day 34) as indicated on the x-axis (**D**). Statistical analysis for C and D was performed by One-way ANOVA (Kruskal-Wallis test followed by Dunn´s post test). Asterisks indicate statistically significant differences (*p<0.05, **p<0.01).

Having shown that T cell-derived IFNγ as well as TNFα activate the bactericidal function of macrophages and inhibit the growth of *R*. *typhi* in infected macrophages *in vitro*, we speculated that TNFα may compensate for the absence of IFNγ in protection against *R*. *typhi* by CD4^+^IFNγ^-/-^ T cells. To test the contribution of TNFα to bacterial killing by CD4^+^IFNγ^-/-^ T cells we isolated naïve and immune CD4^+^ T cells from *R*. *typhi*-infected BALB/c IFNγ^-/-^ mice on day 7 post infection and incubated these cells with *R*. *typhi*-infected macrophages *in vitro*. TNFα was blocked by the addition of neutralizing antibody and bacterial growth was assessed by qPCR. CD4^+^IFNγ^-/-^ T cells indeed inhibited bacterial growth at least by tendency. While 52.91±13.03 *R*. *typhi prsA* gene copies were detectable in cultures containing naïve CD4^+^IFNγ^-/-^ T cells, only 6.55±4.91 *prsA* copies were present in cultures with CD4^+^IFNγ^-/-^ T cells from immune animals. Inhibition of bacterial growth by immune CD4^+^IFNγ^-/-^ T cells was partially restored by the neutralization of TNFα (16.81±13.20 *prsA* copies), indicating a contribution of TNFα to bacterial killing by CD4^+^IFNγ^-/-^ T cells (Fig 7A).

Next, we again adoptively transferred CD4^+^ T cells from IFNγ^-/-^ mice into CB17 SCID mice one day prior to *R*. *typhi* infection and neutralized TNFα to clarify the role of this cytokine in CD4^+^ T cell-mediated protection *in vivo*. Control mice were infected with *R*. *typhi* and received PBS instead of T cells. Mice were then either treated with isotype antibody or with neutralizing anti-TNFα every three days. Weight change, clinical score and survival rates were assessed. All *R*. *typhi*-infected CB17 SCID control mice continuously lost weight, developed a high clinical score and succumbed to the infection within 21 days whether treated with isotype antibody or anti-TNFα (Fig 7B). 2 out of 7 (29%) of the CD4^+^IFNγ^-/-^ T cell recipients that received isotype antibody survived the infection (Fig 7B). The surviving animals of this group lost weight until day 18 and showed a peak clinical score of 3–4 on day 16 post infection. The mice then recovered (Fig 7B). Surprisingly, the survival rate of anti-TNFα-treated CD4^+^IFNγ^-/-^ T cell recipients was higher. 5 out of 7 (86%) of the anti-TNFα-treated CD4^+^IFNγ^-/-^ T cell recipients survived the infection (Fig 7B). Furthermore, disease of these animals was apparently milder and the animals recovered faster. The weight loss of surviving animals of this group was less pronounced and peaked on day 16 post infection (-5.44±2.59%) while isotype-treated surviving CD4^+^IFNγ^-/-^ T cell recipients still showed weight loss of -16.45±1.55% at this point in time (Fig 7B). In addition, the peak of disease as measured by clinical scoring was 4 days earlier (day 12) than that of isotype-treated CD4^+^IFNγ^-/-^ T cell recipients and the animals recovered faster (Fig 7B). All CD4^+^IFNγ^-/-^ T cell recipients that finally succumbed to the infection continuously lost weight and developed a clinical score with similar kinetics or even a little earlier than control mice, whether treated with isotype or anti-TNFα antibody (Fig 7B). These data show that TNFα obviously exerts pathological effects and is non-beneficial in CD4^+^ T cell-mediated protection in the absence of IFNγ.

Because CB17 SCID mice develop severe liver necrosis upon *R*. *typhi* infection [21], we also assessed serum GPT levels as a measure for liver damage in all groups of mice at the time of death and in surviving CD4^+^IFNγ^-/-^ T cell recipients at day 34 post infection when the experiment was terminated. GPT levels in CD4^+^IFNγ^-/-^ T cell recipients including those that succumbed to the infection were generally normal, whether treated with isotype antibody or anti-TNFα, while serum GPT was enhanced in control animals compared to non-infected mice (Fig 7C). Liver damage in control mice was unaltered by the application of anti-TNFα (Fig 7C). These data show that TNFα is generally not involved in liver damage in *R*. *typhi*-infected CB17 SCID mice and that CD4^+^IFNγ^-/-^ T cells completely prevent liver damage in the presence or absence of this cytokine.

To analyze bacterial clearance, we further quantified the bacterial burden of all animals that succumbed to the infection at the time of death and of surviving mice at day 34 when the experiment was terminated. As expected, control CB17 SCID mice that did not receive T cells developed high and comparable bacterial loads in all organs when treated with isotype antibody (spleen: 94547±30121, brain: 11109±3904, lung: 1624±407.6, liver: 1310±292.6 *prsA* copies) (Fig 7D). The application of anti-TNFα did not alter the bacterial load in these animals (spleen: 80536±19501, brain: 12259±2881, lung: 2450±724.8, liver: 828.4±17.1 *prsA* copies) (Fig 7D). Bacterial numbers were reduced in all CD4^+^IFNγ^-/-^ T cell recipients including those that succumbed to the infection. Isotype-treated mice of this group that died upon infection had 278.4±150.3 *prsA* copies in the spleen, 499.2±367.3 copies in the brain, 77.3±29.7 copies in the lung and 103.1±82.6 copies in the liver and the mouse of the anti-TNFα-treated group that died on day 13 also had reduced amounts of bacteria in all organs (spleen: 286, brain: 621.5, lung: 454.5, liver: 456.0 *prsA* copies) (Fig 7D). CD4^+^IFNγ^-/-^ T cell recipients that survived the infection and were treated with isotype antibody were almost negative for *R*. *typhi*. The bacteria were generally not detectable in the liver and lung of these animals while low copy numbers were detectable in the spleen (1.12±1.12 copies) and brain (64.93±19.13 copies) at the end of the experiment. Similar was also true for anti-TNFα-treated CD4^+^IFNγ^-/-^ T cell recipients. Here, spleen and liver were negative for *R*. *typhi* while low copy numbers were found in the brain (14.34±9.51 copies) and lung (18.50±16.30 copies). These data clearly demonstrate that TNFα is not essential for bacterial elimination by CD4^+^IFNγ^-/-^ T cells *in vivo*, and that TNFα exerts pathological effects.

### CD4^+^IFNγ^-/-^ T cells produce T_H_17 cytokines in *R*. *typhi* infection

**Fig 8:**
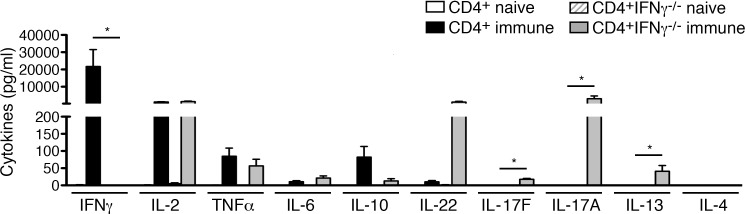
CD4^+^IFNγ^-/-^ differentiate into T_H_17 cells that produce large amounts of IL-17A and IL-22 upon *R*. *typhi*-specific restimulation. 1×10^6^ bone-marrow-derived BALB/c macrophages were infected with 5 copies of *R*. *typhi* per cell one day prior to the addition of 2×10^6^ purified CD4^+^ T cells from either naïve or immune wild-type BALB/c or BALB/c IFNγ^-/-^ mice (day 7 post infection). Cytokines were quantified in the cell culture supernatant by LEGENDplex assay 72h after T cell addition (n = 4 for each group). Statistical analysis of cytokine production by wild-type CD4^+^ and CD4^+^IFNγ^-/-^ T cells was performed by One-way ANOVA (Kruskal Wallis test followed by Dunn´s post test) (*p<0.05).

The previous experiment gave rise to the question which alternative cytokines may then be involved in protection by CD4^+^ T cells in the absence of both IFNγ and TNFα. Therefore, we compared the cytokine profile that is expressed by immune wild-type BALB/c and BALB/c CD4^+^IFNγ^-/-^ T cells upon antigen recognition. Either naïve or immune CD4^+^ T cells prepared from BALB/c and BALB/c IFNγ^-/-^ mice on day 7 post *R*. *typhi* infection were incubated with infected macrophages *in vitro* to achieve antigen-specific restimulation. Naïve CD4^+^ T cells were used as a control. Cytokines were generally not detectable in cultures containing *R*. *typhi*-infected macrophages and naïve CD4^+^ T cells (Fig 8).

Cultures of infected macrophages and immune wild-type CD4^+^ T cells produced very high amounts of IFNγ (21749±9799 pg/ml) in addition to IL-2 (1213±104 pg/ml) and lower amounts of TNFα (84.97±23.68 pg/ml). Furthermore, low levels of IL-10 (82.22±31.18 pg/ml) and negligible amounts of IL-22 (10.37±3.65 pg/ml) and IL-6 (10.71±3.09 pg/ml) were measurable in cultures with immune wild-type CD4^+^ T cells. Other cytokines were not produced (Fig 8).

In contrast, the supernatants of cocultures of immune CD4^+^IFNγ^-/-^ T cells and *R*. *typhi*-infected macrophages contained high amounts of IL-17A (3061±1587 pg/ml) and IL-22 (1127±401 pg/ml) in addition to low levels of IL-17F (17.76±2.35 pg/ml) while only low amounts of IL-13 (41.13±16.76 pg/ml) were detectable. IL-4 was not produced at all (Fig 8). Amounts of IL-2 (1407±314 pg/ml), TNFα (56.65±19.59 pg/ml), IL-10 (12.98±6.50 pg/ml) and IL-6 (21.49±6.14 pg/ml) were comparable to those produced by cultures with immune wild-type CD4^+^ T cells (Fig 8). Thus, immune wild-type CD4^+^ T cells clearly show a T_H_1 cytokine profile characterized by the production of IFNγ and TNFα whereas CD4^+^IFNγ^-/-^ T cells acquire a T_H_17 phenotype, producing IL-17A, IL-22 and TNFα. The T_H_2 response by these cells is negligible.

### The neutralization of IL-17A in *R*. *typhi*-infected CD4^+^IFNγ^-/-^ T cell recipients mitigates disease and increases the probability to survive the infection

**Fig 9:**
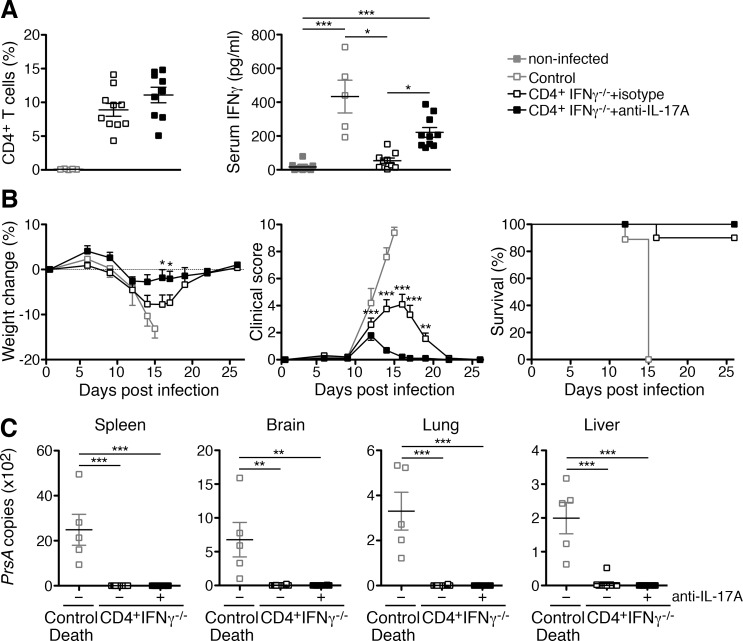
Enhanced protection by CD4^+^IFNγ^-/-^ T cells in the absence of IL-17A. 1×10^6^ purified CD4^+^ T cells from BALB/c IFNγ^-/-^ mice were adoptively transferred into CB17 SCID mice one day prior to the infection with 1×10^6^ sfu *R*. *typhi*. IL-17A was neutralized by intraperitoneal application of 500 μg anti-IL-17A every two days beginning on day 2 post infection (n = 10). A second group of T cell recipients received isotype antibody (n = 10). *R*. *typhi*-infected control mice did not receive T cells and were treated with PBS instead of antibody (n = 5). The CD4^+^ T cell frequency among leukocytes in the blood (y-axis) was determined on day 8 post transfer (left). CD8^+^ T cells were not detectable. Plasma cytokines were detected by LEGENDplex assay. Plasma from naïve CB17 SCID mice (n = 10) was used as a control. Only IFNγ (y-axis) was detectable at significant amounts. Statistical analysis was performed by One-way ANOVA (Kruskal Wallis test followed by Dunn´s post test) (*p<0.05, ***p<0.001) (**A**). The state of health of the mice was monitored by weight change (y-axis, left) and a clinical score (y-axis, middle) and the survival rates (y-axis, right) were assessed. Statistical analysis of survival rates was performed with Log-rank (Mantel-Cox) test. Differences in the survival of anti-IL-17A- and isotype-treated animals were not significant (**B**). The bacterial content (y-axis) in the organs was quantified by *prsA*-specific qPCR from all control animals that succumbed to the infection at the time of death and from all CD4^+^IFNγ^-/-^ T cell recipients including the mouse of the isotype-treated group that died on day 16 post infection. Samples from the surviving animals were taken at the end of the experiment (day 26). Statistical analysis was performed by One-way ANOVA (Kruskal-Wallis test followed by Dunn´s post test). Asterisks indicate statistically significant differences (**p<0.01, ***p<0.001) (**C**).

Because immune CD4^+^IFNγ^-/-^ T cells produce no other cytokines than IL-17A, IL-22 and TNFα at higher amounts, the data presented so far strongly suggest that the T_H_17 cytokines IL-17A and IL-22 are involved in protection by CD4^+^ T cells in the absence of both IFNγ and TNFα. To test this hypothesis, we finally performed adoptive transfer of CD4^+^IFNγ^-/-^ T cells into *R*. *typhi*-infected CB17 SCID mice and treated the animals either with neutralizing anti-IL-17A or isotype antibody. Control mice were infected with *R*. *typhi* and neither received T cells nor antibody. First of all, we analyzed the mice for the presence of T cells in the blood on day 8 post infection. Isotype- and anti-IL-17A-treated CD4^+^IFNγ^-/-^ T cell recipients showed comparable frequencies of CD4^+^ T cells among leukocytes in the blood (isotype: 8.90±0.95%, anti-IL-17A: 11.09±1.15%) (Fig 9A, left).

Second, we analyzed the production of plasma cytokines at the same point in time and included plasma from non-infected CB17 SCID mice as an additional control. In these mice IFNγ was negligible (17.28±7.59 pg/ml) while significantly enhanced amounts of the cytokine were detectable in *R*. *typhi*-infected control animals (433.5±97.08 pg/ml). Compared to these, isotype-treated CD4^+^IFNγ^-/-^ T cell recipients showed strongly reduced plasma levels of IFNγ (isotype: 53.98±47.90 pg/ml) while amounts of the cytokine were only reduced by half in anti-IL-17A-treated mice (221.4±28.93 pg/ml) (Fig 9A, right). These data suggest that IL-17A released by the T cells suppresses the production of IFNγ in CB17 SCID mice where NK cells and macrophages represent the major sources of this cytokine upon *R*. *typhi*-infection [21]. Enhanced amounts of other cytokines were not detectable at this early point in time.

Contrary to our expectations, however, anti-IL-17A-treated CD4^+^IFNγ^-/-^ T cell recipients showed much milder disease than isotype-treated animals. All CD4^+^IFNγ^-/-^ T cell recipients that received anti-IL-17A hardly lost weight upon *R*. *typhi* infection (Fig 9B, left), showed a very weak temporary clinical score peaking on day 12 post infection (Fig 9B, middle) and survived the infection (Fig 9B, right). In contrast, isotype-treated CD4^+^IFNγ^-/-^ T cell recipients significantly lost weight between day 14 and 21 compared to anti-IL-17A-treated animals (Fig 9B, left). In addition, isotype-treated CD4^+^IFNγ^-/-^ T cell recipients developed a significantly higher and prolonged clinical score peaking on day 16 when anti-IL-17A-treated animals already appeared healthy (Fig 9B, middle). Nonetheless, only one mouse of this group died through the infection (Fig 9B, right), demonstrating once more that CD4^+^ T cells are capable of protecting against *R*. *typhi* without the capability to produce IFNγ.

We further analyzed the bacterial content in the organs of all animals by qPCR. As expected, PBS-treated control mice that died upon infection showed high amounts of bacteria in the spleen (2485±688.9 copies) while the bacterial burden was lower in the brain (677.7±255.2 copies), lung (330.3±84.12 copies) and liver (199.7±46.33 copies) (Fig 9C). In contrast, all CD4^+^IFNγ^-/-^ T cell recipients whether treated with isotype antibody or anti-IL-17A were negative for *R*. *typhi* in the organs when the experiment was terminated on day 26 post infection. Furthermore, also the only mouse of the isotype-treated CD4^+^IFNγ^-/-^ T cell recipient group that died upon infection on day 16 had only very low amounts of bacteria predominantly in the liver (52.0 copies) followed by the brain (20.8 copies), lung (6.49 copies) and spleen (1.01 copies) (Fig 9C).

These data demonstrate that IL-17A exerts pathological effects similar to TNFα, and that this cytokine is also not essential for bacterial elimination by CD4^+^IFNγ^-/-^ T cells *in vivo*.

## Discussion

In the current study we describe the T cell response in *R*. *typhi*-infected BALB/c mice and show by adoptive transfer into congenic CB17 SCID mice, a lethal model of *R*. *typhi* infection [21], that both CD8^+^ and CD4^+^ T cells are protective, eliminate the bacteria and provide long-term control. The cytotoxic function is not essential for CD8^+^ T cell-mediated protection. Our results further suggest that CD4^+^ T cells require either IFNγ, TNFα or T_H_17 cytokines to activate the bactericidal activity of macrophages and to mediate protection.

*R*. *typhi*-infected BALB/c wild-type mice developed IFNγ-expressing CD4^+^ T cells and cytotoxic CD8^+^ T cells that expressed IFNγ and Granzyme B, an effector molecule that is involved in target cell killing [39]. CD8^+^ T cells in *R*. *typhi*-infected BALB/c mice expressed CD11a and KLRG1, demonstrating that they were functional antigen-experienced effector cells. Both the CD4^+^ and CD8^+^ T cell response peaked on day 7 post infection and then declined as it was also observed in *R*. *typhi*-infected C57BL/6 mice [19]and C3H/HeN mice [40]. Surprisingly, in BALB/c mice activated T cells were again detectable late in infection at day 35. This observation is interesting as we could recently show that *R*. *typhi* persists in BALB/c as well as in C57BL/6 wild-type mice [20]. Reactivation of T cells, however, was not observed in *R*. *typhi*-infected C57BL/6 mice that instead seem to maintain a certain level of effector cells until day 35 [19]. Comparing the T cell response of BALB/c and C57BL/6 mice it is obvious that both the activation of cytotoxic CD8^+^ T effector cells and IFNγ-expressing CD4^+^ T cells both of which are important for the control of intracellular pathogens is less efficient in animals of the BALB/c background upon *R*. *typhi* infection. For example, 5% of the CD8^+^ T cells expressed Granzyme B in *R*. *typhi*-infected BALB/c mice at the peak of response on day 7 while 10% of the CD8^+^ T cells were Granzyme B^+^ in C57BL/6 mice at this point in time [19]. Similar was true for the CD4^+^ T cell effector response. Approximately 8% of the CD4^+^ T cells in BALB/c mice expressed IFNγ on day 7 whereas 14% of the CD4^+^ T cells were IFNγ^+^ in C57BL/6 mice [19]. Less efficient induction of T_H_1 and cytotoxic T cells in BALB/c mice compared to C57BL/6 mice has been also described for other infections such as *Leishmania major* [41]. The weaker T cell response could be the reason why sporadic reactivation of T cells is necessary to control persisting *R*. *typhi* in BALB/c mice while the continuous presence of low numbers of activated T cells is sufficient to control the bacteria in C57BL/6 mice.

Adoptively transferred CD8^+^ T cells efficiently and quickly eliminated the bacteria in *R*. *typhi*-infected CB17 SCID mice. The bacteria were almost completely eradicated in the organs already by day 7 post infection in CD8^+^ T cell recipients. Furthermore, these animals were completely protected from *R*. *typhi*-induced disease. This was also true for CD8^+^ T cells that either lacked Perforin, an essential component of the cytotoxic machinery [39], or IFNγ. Similar observations were made for the infection of C57BL/6 IFNγ^-/-^ mice with *R*. *australis*, a member of the transitional group. Here, adoptive transfer of immune CD8^+^IFNγ^-/-^ T cells into *R*. *australis*-infected C57BL/6 IFNγ^-/-^ mice was protective and reduced the bacterial load [15]. In the CB17 SCID model of *R*. *typhi* infection CD8^+^Perforin^-/-^ as well as CD8^+^IFNγ^-/-^ T cell recipients remained asymptomatic for a long period of time (>120 days). However, low numbers of *R*. *typhi* were again detectable in the organs of animals that had received CD8^+^IFNγ^-/-^ T cells beyond day 120 but not in CD8^+^Perforin^-/-^ T cell recipients. In this context, it is interesting that the bacteria were predominantly detectable in the brain which may indicate that *R*. *typhi* preferentially persists in this immune privileged organ. It has been suggested that the cytotoxic activity of CD8^+^ T cells is more important in defense against rickettsiae than the production of IFNγ. For example, C57BL/6 Perforin^-/-^ mice showed a higher susceptibility and lethality upon infection with *R*. *australis* compared to C57BL/6 IFNγ^-/-^ mice [15]. Our results, however, suggest that the release of IFNγ is not only sufficient for CD8^+^ T cell-mediated protection against TG rickettsiae but may be even more important than the cytotoxic activity, at least for long-term control of the bacteria.

IFNγ activates the bactericidal activity of macrophages and endothelial cells by inducing the expression of iNOS and subsequent release of NO [31, 32, 42]. Both endothelial cells and macrophages are target cells of rickettsiae [1, 43, 44] that can eliminate the bacteria with the help of IFNγ. CD4^+^ T cells from *R*. *typhi*-infected BALB/c mice produced very high amounts of IFNγ upon antigen-specific restimulation. Similar to CD8^+^ T cells the adoptive transfer of CD4^+^ T cells into *R*. *typhi*-infected CB17 SCID mice was protective. In contrast to CD8^+^ T cell recipients, however, CB17 SCID mice that received CD4^+^ T cells showed temporary signs of disease and enhanced serum GPT levels with similar kinetics as *R*. *typhi*-infected control animals. This observation corresponds with the prolonged presence of *R*. *typhi* in the organs of these mice compared to CD8^+^ T cell recipients. While the bacteria were almost completely eradicated by CD8^+^ T cells on day 7 post infection, the bacterial load in the organs of CD4^+^ T cell recipients was reduced by trend compared to that of control animals at this point in time. However, CD4^+^ T cell recipients then completely recovered from *R*. *typhi*-induced disease and appeared healthy by day 21 suggesting bacterial elimination within this time frame. Moreover, although CD4^+^ T cells were obviously less efficient than CD8^+^ in bacterial elimination, they provided long-term protection against recurrence of *R*. *typhi*. The bacteria were detectable at very low levels in only very few mice beyond day 120 post infection. Thus, CD4^+^ T cells are sufficient to protect CB17 SCID mice from *R*. *typhi*. In line with these observations, immune CD4^+^ T cells were protective against a lethal infection of C3H/HeN mice with *R*. *conorii* [14] and of C57BL/6 RAG1^-/-^ mice with *R*. *typhi* [19].

We further show that CD4^+^ T cells act bactericidally and inhibit bacterial growth in infected macrophages *in vitro*. CD4^+^ T cells may acquire cytotoxic function during *R*. *typhi* infection *in vivo*. For example, cytotoxic CD4^+^ T cells are observed in chronic viral infections of humans, e.g. cytomegalovirus (HCMV), hepatitis virus and human immunodeficiency virus 1 (HIV-1) [45–51] and mice, e.g. lymphocytic choriomeningitis virus (LCMV) and gamma-herpes virus [52, 53]. These cells are characterized by the upregulation of CD11a and the expression of Granzyme B [30]. Both markers were not detectable in CD4^+^ T cells from *R*. *typhi*-infected mice. Therefore, it is unlikely that immune CD4^+^ T cells from *R*. *typhi*-infected BALB/c mice exert direct cytotoxicity. CD4^+^ T cells most likely mediate bacterial elimination by macrophages via the induction of bactericidal mechanisms via cytokines. Immune CD4^+^ T cells from *R*. *typhi*-infected BALB/c mice induced the release of NO by *R*. *typhi*-infected macrophages *in vitro* and produced high amounts of antigen-specific IFNγ and lower amounts of TNFα *in vitro* and *in vivo* upon transfer into *R*. *typhi*-infected CB17 SCID mice. Similar to IFNγ, also TNFα induces the expression of iNOS in MΦ [31] and synergizes with IFNγ in this effect [36]. Correspondingly, NO release induced by immune CD4^+^ T cells from BALB/c wild-type mice was inhibited by the addition of either neutralizing IFNγ- or TNFα-specific antibody, demonstrating that this effect depends in large part on these cytokines. Moreover, recombinant IFNγ and TNFα alone strongly reduced the growth of *R*. *typhi* in infected macrophages *in vitro*, showing the bactericidal effect of both cytokines. Nonetheless, bacterial growth in the presence of immune CD4^+^ T cells was not completely restored by neutralization of IFNγ or TNFα. This can be explained, however, by incomplete neutralization of the cytokines.

Both IFNγ and TNFα have been shown to be important in defense against a number of intracellular pathogens. TNFα enhances the phagocytic activity and helps macrophages to control intracellular growth of *Mycobacterium* (*M*.) *tuberculosis* [54]. IFNγ further induces the expression of TNFα in macrophages which is required for IFNγ-mediated induction of iNOS [55], and coactivation by IFNγ and TNFα is needed for the induction of full bactericidal activity and granuloma formation in *M*. *tuberculosis* infection [56]. Similarly, IFNγ and TNFα were found to be important in rickettsial defense. Neutralization of either IFNγ or TNFα leads to enhanced bacterial growth and fatality in *R*. *conorii*-infected C3H/HeN mice [17]. Second, C57BL/6 IFNγ^-/-^ mice show enhanced susceptibility and lethality in the infection with *R*. *australis* [15]. It has been assumed that TNFα produced by macrophages acts in a synergistic manner with T cell- and NK cell-derived IFNγ on adjacent infected cells such as endothelial cells, hepatocytes and macrophages to induce NO production and rickettsial killing [17]. Indeed, we found that macrophages express TNFα in *R*. *typhi*-infected CB17 SCID mice. In addition, NK cells as well as macrophages produce IFNγ in these animals upon *R*. *typhi* infection [21]. Nonetheless, the high amounts of IFNγ and TNFα that are released by NK cells and macrophages in CB17 SCID mice upon *R*. *typhi* infection are not protective but rather seem to be the reason for death [21]. Together with the findings from the current study these observations suggest that IFNγ and TNFα must be locally provided by T cells to act bactericidal, and that both cytokines are involved in protection mediated by IFNγ-competent CD4^+^ T cells.

Because IFNγ is considered the most important cytokine involved in protection against intracellular pathogens including rickettsiae, it was then surprising that this cytokine is obviously not essential for protection against *R*. *typhi*. BALB/c IFNγ^-/-^ mice infected with *R*. *typhi* not only survived the infection but were absolutely asymptomatic. Moreover, even adoptive transfer of CD4^+^ T cells that lack IFNγ still protected 30–90% of the *R*. *typhi*-infected CB17 SCID mice. Bacterial numbers in the organs even of those CD4^+^IFNγ^-/-^ T cell recipients that succumbed to the infection were clearly reduced at the time of death compared to *R*. *typhi*-infected control mice, demonstrating bactericidal activity of CD4^+^IFNγ^-/-^ T cells *in vivo*. Furthermore, CD4^+^IFNγ^-/-^ T cells provided long-term control in those animals that survived the infection. Only very low copy numbers of *R*. *typhi* were detectable in few animals late post infection (> d120). Although harboring persistent bacteria these mice appeared healthy. Finally, CD4^+^IFNγ^-/-^ T cells inhibited the growth of *R*. *typhi* in infected macrophages *in vitro*. Similar to immune CD4^+^ T cells from wild-type mice, this effect was inhibited at least in part by the neutralization of TNFα. Thus, CD4^+^ T cells lacking IFNγ obviously exert bactericidal functions and are clearly able to eliminate and control *R*. *typhi* by activating macrophages. This may be in part mediated by TNFα.

The neutralization of TNFα in *R*. *typhi*-infected CD4^+^IFNγ^-/-^ T cell recipients, however, led to milder disease and enhanced survival, and the cells were still capable of eliminating the bacteria. These findings demonstrate pathological effects of TNFα that were not observed in mice that received wild-type T_H_1 cells, and that other factors, most likely IL-17A and IL-22, play a dominant role in protection in this situation. In contrast to immune CD4^+^ T cells from wild-type mice, CD4^+^IFNγ^-/-^ T cells produced high amounts of these cytokines in addition to lower levels of IL-17F instead of IFNγ upon antigen recognition. IL-2 and TNFα were produced at equal amounts by wild-type CD4^+^ and CD4^+^IFNγ^-/-^ T cells and negligible amounts of T_H_2 cytokines were released. Other cytokines such as IL-21 that could be involved in bacterial defense were not detectable. IL-17A and F were not produced at all by immune wild-type CD4^+^ T cells and amounts of IL-22 were very low. These data show that CD4^+^ T cells preferentially differentiate to T_H_17 cells that are characterized by the production of IL-17A, IL-17F and IL-22 [57] in the absence of IFNγ.

T_H_17 cells have been mainly involved in protective immunity against extracellular bacterial pathogens such as *Klebsiella pneumonia* and *Citrobacter rodentium* [58–60]. In recent years, however, T_H_17 cells have also been implicated in defense against intracellular bacteria and parasites. IL-17A and F mediate in part similar biological effects [61, 62] and induce the activation of NF-κB [63–65] and the production of granulopoietic factors such as G-CSF, GM-CSF and stem cell factor and chemokines (CXCL-1, CXCL-2, CXCL-5, CXCL-8) that are involved in the recruitment of neutrophils [57]. These can contribute to parasite elimination. Apart from that, IL-17A/F induce the release of inflammatory cytokines (TNFα, IL-6, IL-1β) and matrix metalloproteases (MMPs) in tissue cells and macrophages [66–70] and of antimicrobial peptides in tissue cells in concert with IL-22 [71, 72]. In addition, both IL-17A and IL-22 exert direct antiparasitic effects in target cells. IL-17A reduces the growth of the intracellular bacterium *Chlamydia* (*C*.) *muridarum* in the lung of infected mice *in vivo* and in lung epithelial cells and macrophages *in vitro* in an iNOS-dependent manner [73]. IL-17A promotes IFNγ-induced expression of iNOS and NO release in lung epithelial cells *in vivo* and acts protective in the infection with *C*. *muridarum* [73]. In line with these findings, IL-17A was found to promote the expression of iNOS and the release of NO by macrophages infected with *Mycobacterium bovis* bacillus Calmette-Guerin (BCG) [74]. In addition, IL-17A directly inhibits the growth of the intracellular parasite *Trypanosoma* (*T*.) *cruzi* in infected macrophages *in vitro* [75]. Here, killing of intracellular *T*. *cruzi* parasites is mediated via the activation of the NAPDH oxidase [75] that produces superoxide and other reactive oxygen species (ROS) that can mediate killing of intracellular pathogens in macrophages and neutrophils similar to NO [76]. A protective activity of T_H_17 cells was further demonstrated in the infection with *T*. *cruzi in vivo*. Adoptive transfer of *T*. *cruzi*-specific TCR-transgenic T_H_17 cells into C57BL/6 RAG1^-/-^ mice protects the animals against a lethal challenge with *T*. *cruzi* [75]. Finally, treatment of epithelial cells infected with the intracellular apicomplexan parasite *Eimeria* (*E*.) *falciformis* with either IFNγ, IL-22 or IL-17A reduces parasite growth *in vitro* [77]. Moreover, C57BL/6 IFNγR^-/-^ as well as C57BL/6 IFNγ^-/-^ mice infected with this parasite showed higher pathology and body weight loss but reduced pathogen burden compared to wild-type mice [77], demonstrating that IFNγ is dispensable for the elimination of this pathogen. As observed for CD4^+^ T cells in the infection of BALB/c IFNγ^-/-^ mice with *R*. *typhi*, the infection of C57BL/6 IFNγ^-/-^ mice with *E*. *falciformis* was also associated with increased production of IL-17A and IL-22 by CD4^+^ T cells, and the authors further show that the neutralization of both IL-22 and IL-17A or IL-22 alone in C57BL/6 IFNγR^-/-^ mice leads to increased parasite load *in vivo* [77], indicating a dominant protective effect of IL-22 in this infection.

Surprisingly, the neutralization of IL-17A in *R*. *typhi*-infected CD4^+^IFNγ^-/-^ recipient mice led to a very similar outcome as the neutralization of TNFα (milder disease, reduced body weight loss and increased probability to survive the infection), and the animals were still capable of eliminating the bacteria. These findings demonstrate pathological effects of IL-17A. In this context, it is interesting that the neutralization of both IL-17A and IL-22 but not one or the other cytokine alone in *E*. *falciformis*-infected C57BL/6 IFNγR^-/-^ mice abolished body weight loss [77]. This indicates pathological effects of IL-17A as well as of IL-22 that have been also implicated in other situations such as progressive airway inflammation in a murine model of bleomycin-induced airway inflammation [78]. As mentioned before, IL-17A induces the release of inflammatory cytokines such as IL-6, TNFα and IL-1β [66–70]. IL-17A may further synergize with these mediators to aggravate tissue inflammation and damage [79, 80] which is strongly supported by our observations that suggest synergistic pathological effects of TNFα and IL-17A in *R*. *typhi*-infected mice. On the contrary, the release of IL-22 in combination with either TNFα or IL-17A is beneficial and sufficient to mediate protection. We suggest that both IL-17A and TNFα directly act on infected cells such as macrophages and neutrophils similar to IFNγ, activating the bactericidal activity but also inducing inflammatory mediators, while IL-22 may support bacterial elimination by the induction of antimicrobial peptides and other factors in infected non-immune cells such as fibroblasts and endothelial cells.

Whether T_H_17 cells are as effective in bacterial elimination as T_H_1 cells is not clear. The observation that surviving CD4^+^IFNγ^-/-^ recipients showed prolonged disease compared to animals that received wild-type CD4^+^ T cells may argue for a more effective bacterial killing by wild-type T_H_1 cells and prolonged persistence of *R*. *typhi*. Nevertheless, also CD4^+^IFNγ^-/-^ T cell recipients were almost free of bacteria at the time of death. In addition, the course of disease in CD4^+^IFNγ^-/-^ T cell recipients after neutralization of either TNFα or IL-17A was mild and comparable to that of animals that had received IFNγ-competent CD4^+^ T cells. Therefore, a more likely explanation for prolonged disease in CD4^+^IFNγ^-/-^ T cell recipients is the induction of enhanced pathology due to the combined release of TNFα and IL-17A by CD4^+^IFNγ^-/-^ T cells. The exact mechanisms how T_H_17 cytokines mediate protection and/or pathology in *R*. *typhi* infection remain to be elucidated. At least, we can conclude that IFN© which is produced by innate immune cells in *R*. *typhi*-infected CB17 SCID mice [21] is not involved. The production of IFNγ was not significantly altered upon IL-17A neutralization and reduced rather than enhanced in CD4^+^IFNγ^-/-^ T cell recipients compared to control animals. Moreover, IFNγ was clearly reduced in isotype-treated CD4^+^IFNγ^-/-^ T cell recipients, arguing against a contribution of innate-derived IFNγ to bacterial elimination and pathology.

Collectively, we show that the cytotoxic activity of CD8^+^ T cells is not essential for protection against *R*. *typhi* while the release of IFNγ by CD8^+^ T cells seems to be even more important than the cytolytic function for long-term control of the bacteria. We further show that IFNγ-producing CD4^+^ T_H_1 as well as T_H_17 cells that release TNFα, IL-17A and IL-22 protect CB17 SCID mice against *R*. *typhi*, most likely by activating the bactericidal activity of macrophages, and that the combined production of TNFα and IL-17A exerts non-beneficial immunopathologic effects.
